# Familial association of pseudohypoparathyroidism and psoriasis: case report

**DOI:** 10.1590/S1516-31802002000100007

**Published:** 2002-01-02

**Authors:** Renan Magalhães Montenegro, Francisco José Albuquerque de Paula, Norma Tiraboshi Foss, Milton Cesar Foss

**Keywords:** Pseudohypoparathyroidism, Hypocalcemia, Albright osteodystrophy, Psoriasis, Pseudo-hipoparatiroidismo, Hipocalcemia, Osteodistrofia de Albright, Psoríase

## Abstract

**CONTEXT:**

The association between psoriasis and hypoparathyroidism has been reported by several authors, and it has been suggested that abnormalities in calcium homeostasis may be involved in the development or exacerbation of psoriasis. However, so far there have only been two reports of pseudohypoparathyroidism associated with psoriasis.

**OBJECTIVE:**

To describe the familial occurrence of this association for the first time.

**CASE REPORTS:**

Two siblings with psoriasis associated with pseudohypoparathyroidism were presented. The first patient was a 24-year-old white male with disseminated erythrodermic pustular psoriasis that began 2 months before admission. He had had a history of mental retardation, recurrent otitis, seizures and arthralgia from the age of 11 years onwards. He presented the characteristic phenotype of Albright osteodystrophy: short stature, obesity, round facies, broad forehead, short neck and brachydactylia. He adopted a position of flexed limbs and showed proximal muscle weakness and a positive Trousseau sign. He had clinical signs of hypocalcemia (0.69 mmol/l ionized calcium and 3.2 mg/dl total calcium), hyperphosphatemia (6.6 mg/dl), hypomagnesemia (1.0 mEq/l), hypoalbuminemia (3.1 g/dl), normal serum intact PTH levels (45.1 pg/ml), primary hypothyroidism (13.2 mU/ml TSH, and 4.7 mg/dl total T_4_), hypergonadotrophic hypogonadism (116.0 ng/ml LH, 13.2 mU/ml FSH and 325.0 ng/dl testosterone), osteoporosis, and diffuse calcifications in soft tissues and in the central nervous system. The second case was a 14-year-old white girl with a history of psoriasis vulgaris from the age of five years onwards, and antecedents of mental retardation. She presented signs of Albright osteodystrophy (short stature, round facies, obesity, short neck, brachydactylia), hypocalcemia (ionized calcium of 1.08 mmol/l and total calcium of 6.7 mg/dl) hyperphosphatemia (9.4 mg/dl), elevated serum PTH levels (223.0 pg/ml), osteoporosis, and hypergonadotrophic hypogonadism (7.0 mU/ml LH, 9.3 mU/ml FSH and undetectable estradiol levels).

## RESUMO

**CONTEXTO:**

A associação entre psoríase e hipoparatiroidismo tem sido descrita por vários autores, sendo sugerido que anormalidades na homeostase do cálcio possam estar envolvidas no desenvolvimento ou exacerbação da psoríase. Entretanto, até esse momento, há apenas dois relatos de pseudohipoparatiroidismo em associação com psoríase.

**OBJETIVO:**

Descrever pela primeira vez a ocorrência familiar dessa associação.

**RELATO DE CASO:**

Apresentamos dois irmãos com psoríase associada ao pseudo-hipoparatiroidismo. O primeiro é um paciente de 24 anos, masculino, branco, com psoríase pustulosa eritrodérmica disseminada, de início 2 meses antes da admissão. Tinha antecedentes de retardo neuropsicomotor, otites de repetição, convulsões e artralgias desde os 11 anos de idade. Apresentava fenótipo característico de Osteodistrofia de Albright - baixa estatura, obesidade, fácies arredondada, fronte ampla, pescoço curto, braquidactilia e sinais clínicos de hipocalcemia (adotava posição de flexão dos membros, tinha fraqueza muscular proximal e sinal de Trosseau positivo), hipocalcemia (cálcio ionizado de 0.69 mmol/l e total de 3,2 mg/dl), hiperfosfatemia (6,6 mg/dl), hipomagnesemia (1,0 mEq/l), hipoalbuminemia (3,1 g/dl), níveis séricos de PTH (molécula intacta) normais (45,1 pg/ml), hipotiroidismo primário (TSH de 12,2 mU/ml, e T_4_ total de 4,7 mg/dl), hipogonadismo hipergonadotrófico (LH de 116,0 ng/ml, FSH de 13,2 mU/ml e testosterona de 325,0 ng/dl), osteoporose, calcificações difusas em partes moles e no sistema nervoso central. O segundo caso é de uma paciente de 14 anos, feminina, branca, irmã do 1° paciente, com história de psoríase vulgar desde os 5 anos de idade e antecedentes de retardo neuro-psicomotor. Apresentava sinais da Osteodistrofia de Albright (baixa estatura, obesidade, arredondada, pescoço curto, braquidactilia), hipocalcemia (cálcio ionizado de 1,08 mmol/l e total de 6,7 mg/dl) hiperfosfatemia (9,4 mg/dl), níveis séricos de PTH elevados (222,0 pg/ml), osteoporose e hipogonadismo hipergonadotrófico (LH de 7,0 mU/ml, FSH de 9,3 mU/ml e níveis indetectáveis de estradiol).

## INTRODUCTION

Psoriasis is a skin disease of unknown etiology with prevalence of 1 to 3%, characterized by increased epidermal proliferation and decreased cell turnover.^1^ Abnormalities in calcium homeostasis have been implicated in the pathogenesis of psoriasis. Experimental and clinical vitamin D deficiency has been associated with psoriasis.^1,2^ The effectiveness of vitamin D analogs in the treatment of psoriasis has also favored this hypothesis, although psoriasis may be related to the paracrine actions of 1.25(OH)_2_D.^3^

The association of psoriasis with hypocalcemia has previously been described by several authors.^4-9^ However, in most cases the metabolic disturbance was secondary to hypoparathyroidism. In these reports, the normalization of calcium levels via administration of calcium and vitamin D resulted in an improvement in the dermatological condition, suggesting that changes in calcemia may be implicated in the development or exacerbation of psoriasis.

Reports of less frequent etiologies of psoriasis-associated hypocalcemia are only to be found in Laymon and Zelickson,^10^ describing psoriasiform plaques in a patient with pseudohypoparathyroidism, and in another description of a girl with pseudohypoparathyroidism and psoriasis vulgaris.^8^

In the present study, we describe for the first time two siblings presenting this rare association of pseudohypoparathyroidism and psoriasis.

## CASE REPORTS

### Patient 1

**Figure 1:**
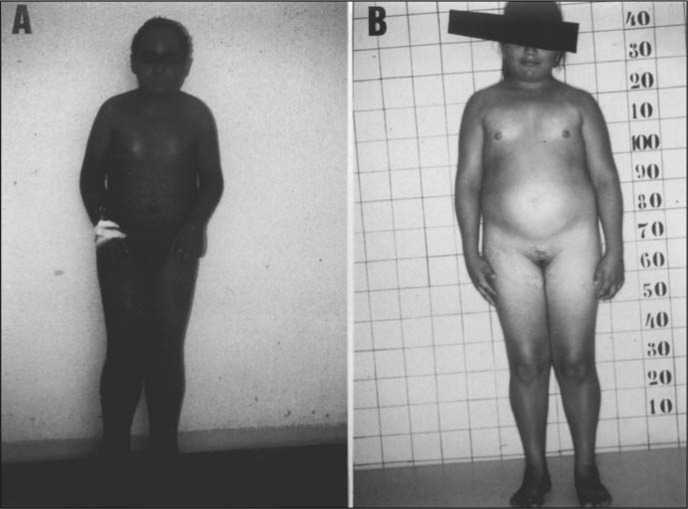
A - Patient 1 before treatment; B - Patient 2 before treatment.

A 24-year-old white male (Figure 1A) reported a history of erythematous scaly lesions in the armpits and inguinal area, which began 2 months before admission, becoming widespread thereafter. He had antecedents of mental retardation, with recurrent otitis, seizures and arthralgia in knees and elbows from the age of 11 years onwards.

**Figure 2:**
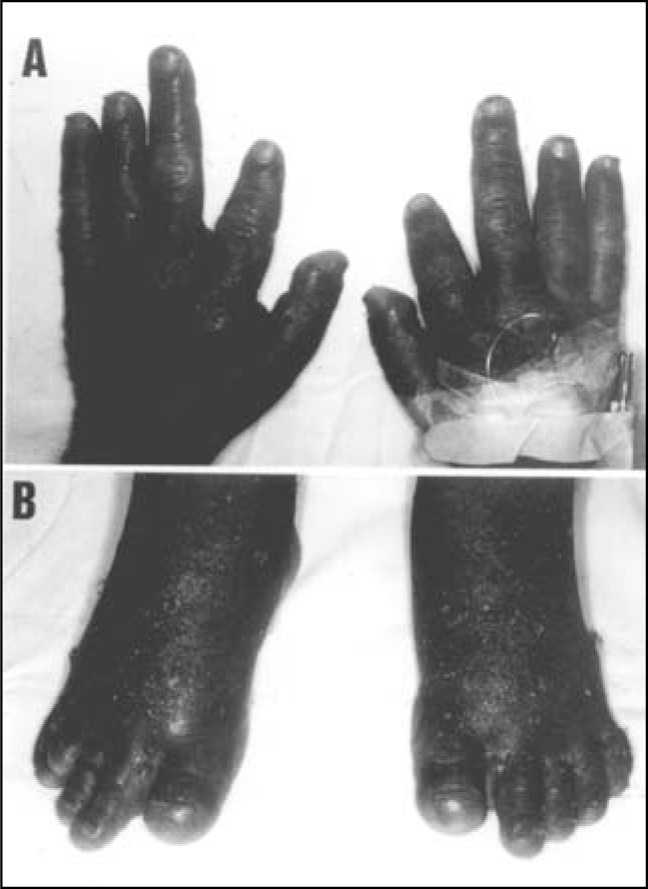
Hands and feet of patient 1.

Physical examination showed: stature of 155 cm; weight of 61.5 kg; body mass index (BMI) of 25.6 kg/m^2^; slight central subcutaneous distribution of adiposity; round facies; broad forehead; short neck; disseminated erythematous scaly plaques; frontal parietal baldness; bilateral shortening of second and fourth fingers, first left and first and fourth right toes (Figure 2); symmetric bilateral edema in the legs; positive Trousseau and negative Chvosteck signs. He had male genitalia of G_4_P_5_ pubertal stage. He presented proximal muscular weakness and difficulty in walking and he adopted a flexed limb position.

Biochemical determinations revealed resistance to thyrotrophic, gonadotrophic and parathyroid hormones (Table). A skin biopsy was compatible with pustular psoriasis. The electroencephalogram revealed nonspecific abnormalities. Sinus tachycardia (120 bpm) and ventricular repolarization disturbances were observed in the electrocardiogram.

**Table:** Biochemical data of the patients (before treatment)

	*Patient 1*	*Patient 2*	*Reference Values*
*Total calcium, mg/dl.*	*3.2*	*6.7*	*8.5 to 10.5*
*Ionized calcium, mmol/l.*	*0.69*	*1.08*	*1.14 to 1.29*
*Inorganic phosphorus, mg/dl.*	*6.6*	*9.4*	*3.7 to 4.5*
*Magnesium, mEq/l.*	*1.0*	*-*	*1.6 to 3.5*
*Alkaline phosphatase, U/l.*	*529.0*	*544.0*	*33.0 to 123.0*
*Albumin, g/dl.*	*3.1*	*4.7*	*3.0 to 5.0*
*Ureic nitrogen, mg/dl.*	*24.0*	*39.0*	*10 to 50*
*Creatinine, mg/dl.*	*0.8*	*0.8*	*0.7 to 1.2*
*Intact PTH, pg/ml.*	*45.1*	*223.0*	*10.0 to 65.0*
*TSH, mU/ml.*	*13.2*	*0.4*	*0.3 to 5.0*
*T_4_,mg/dl.*	*4.7*	*5.4*	*4.9 to 13.9*
*LH, ng/ml.*	*116.0*	*57.4*	*M: 15.0 to 60.0* *F: 10.0 to 40.0*
*FSH, mU/ml.*	*13.2*	*15.4*	*5.0 to 20.0*
*Testosterone, ng/dl.*	*325.0*	*-*	*M: 250.0 to 900.0*
*Estradiol, pg/ml.*	*-*	*36.8*	*F: 30.0 to 350.0*
*Anti-thyroid antibodies*	*negative*	*-*	*< 1:100*

***M: males; F: females***

X-rays of hands and feet showed diffuse osteoporosis, more accentuated in the joints; diffuse micro-calcifications in soft tissue; bilateral shortening of the second and fourth metacarpal bones and the first left and first and fourth right metatarsal bones. A skull Xray showed opened sutures and base ganglion calcifications. Computed tomography of the skull revealed bilateral symmetrical gross diffuse calcifications in the cerebellar hemisphere, base nuclei and thalamus, and in the white and gray matter transition in the frontal, temporal, parietal and occipital regions. Skin lesions did not improve with etretinate.

After the introduction of calcium, vitamin D_3_, magnesium sulfate, and hydrochlorothiazide, there was remission of the cutaneous manifestations (Figure 4), with no recurrence of skin lesions occurring thereafter, although he still presented periods of hypocalcemia due to lack of treatment compliance. Two months later thyroxine was introduced due to a diagnosis of hypothyroidism (Table). Three years later he suddenly died at home of an unknown cause, which was assumed to be related to hypocalcemia.

### Patient 2

A 14-year-old white girl (Figure 1B), the half-sister of the first patient on the mother's side, presented with a history of recurrent diffuse erythematous scaling skin lesions from the age of five years onwards. She had antecedents of mental retardation. Menarche occurred at 13 years of age.

**Figure 3:**
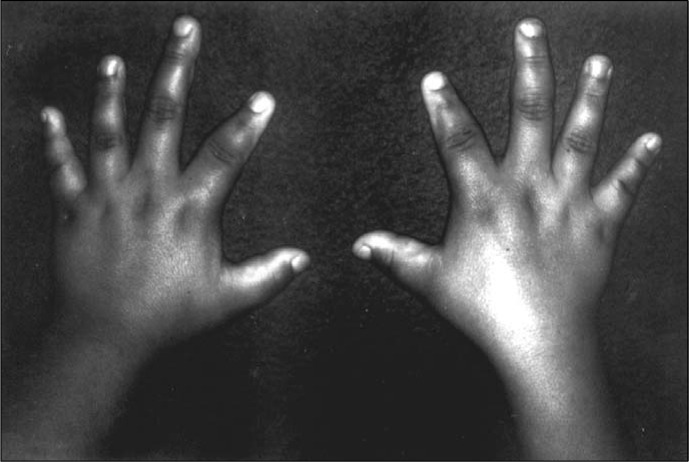
Hands of Patient 2.

Physical examination showed: stature of 147 cm; weight of 60 kg; BMI of 27.8 kg/m^2^; slight central subcutaneous distribution of adiposity; erythematous scaly plaques on the elbows, umbilical area, perineum and legs; round and wide face; short neck; bilateral shortening of first, second and fourth fingers (Figure 3), and first and fourth right toes; and negative Trousseau and Chvosteck signs. She had female genitalia, M_2_ P_5_ pubertal stage.

**Figure 4:**
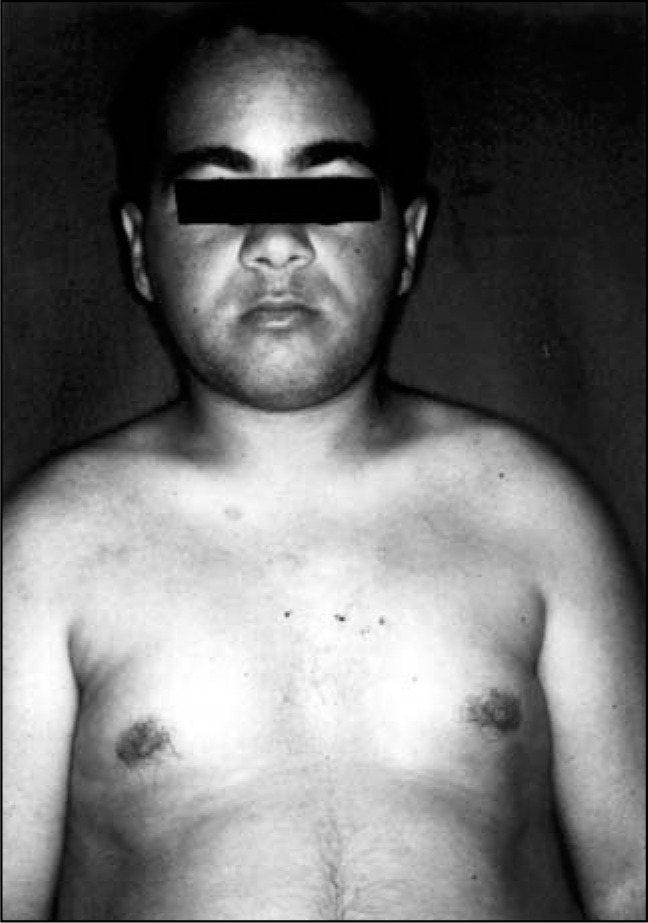
Patient 1 after treatment.

Biochemical determinations revealed parathyroid hormone resistance with normal thyroid and gonadal function (Table). A skin biopsy was compatible with psoriasis vulgaris.

X-rays of hands and feet showed osteoporosis; bilateral shortening of first and fourth metacarpal bones and second proximal phalange bones in the hands, and of the first and fourth right metatarsal bones; bilateral base deformity of second and fifth proximal phalanges in the hands; and phalanx epiphysis coalition. A skull X-ray showed microbrachycephaly, with no pathological calcifications.

Treatment with calcium carbonate and vitamin D_3_ was initiated, but good metabolic control was rarely achieved due to poor treatment compliance. No improvement or exacerbation of skin lesions has occurred since then. At 15 years of age, she started to present amenorrhea with undetectable estradiol levels, 7.0 mU/ml LH and 9.3 mU/ml FSH. A GnRH test confirmed the diagnosis of hypergonadotrophic hypogonadism. A sestamibi ^99m^Tc scan revealed parathyroid hyperplasia.

## DISCUSSION

In the present study, we have described an unusual association of psoriasis and Albright osteodystrophy, which peculiarly affected 2 siblings.

The term pseudohypoparathyroidism describes a group of disturbances characterized by increased PTH levels, hypocalcemia, hyperphosphatemia, and target tissue unresponsiveness to the biological actions of PTH.^11^ Four pseudohypoparathyroidism variants have been described: type Ia, type Ib, type Ic, and type II. Besides the biochemical abnormalities, type Ia pseudohypoparathyroidism is characterized by Albright osteodystrophy phenotype (low stature, obesity, round face, short neck, brachydactylia, cutaneous ossification, mental retardation, and dental hypoplasia). Decreased Gα_S_ protein activity or expression is found in the cell membranes of these patients, secondary to loss of function mutations. In addition to PTH resistance, these patients can display resistance to the action of other hormones. A normocalcemic variant genetically related to this form is known as pseudo-pseudohypoparathyroidism.

Pseudohypoparathyroidism type Ib lacks Albright osteodystrophy manifestations and has normal Gα_S_ protein activity. Pseudo-hypoparathyroidism type Ic clinically and biochemically resembles pseudohypoparathyroidism type Ia (Albright osteodystrophy phenotype and multiple hormone resistance), with the absence of demonstrable defects in Gα_S_ protein activity. Pseudohypoparathyroidism type II has no defined genetic basis. It is characterized by the absence of Albright osteodystrophy manifestations, a normal increase in urinary excretion of nephrogenic cAMP and a reduced phosphaturic effect in response to PTH.^12^

Our patients had the clinical and biochemical phenotype characteristic of Albright osteodystrophy. Therefore, the diagnostic possibilities in these cases would be pseudohypoparathyroidism types Ia or Ic. The former is the most common form of pseudohypoparathyroidism. In the present case, the occurrence of pseudohypoparathyroidism in two siblings with different fathers supports the hypothesis of maternal inheritance of this disease. Paternal inheritance has been suggested to result in pseudo-pseudohypoparathyroidism.^13^

The elevated PTH levels found in patient 2 are compatible with the diagnosis of pseudohypoparathyroidism. Patient 1 presented PTH levels within the normal range. However, this fact could be attributed to the hypomagnesemia detected in this patient, since chronically reduced magnesium levels inhibit PTH secretion.^14^

The two siblings also had hypergonadotrophic hypogonadism. The gonadotrophin and testosterone levels of patient 1 and the pubertal stage he reached suggest a partial and/or late onset disorder. Similarly, patient 2 developed amenorrhea at 15 years of age. However, hypothyroidism and cutaneous calcifications were only observed in patient 1. As observed in these cases, phenotypic expression of Albright osteodystrophy is variable, even in patients from the same family.

An association between psoriasis and hypocalcemia has previously been described by several authors. However, in most cases hypocalcemia was secondary to hypoparathyroidism.^4-9^ Only two cases of pseudohypoparathyroidism associated with psoriasis have been described thus far.^8,10^ In the cited reports, emphasis was placed on the temporary relationship between calcium levels and skin abnormalities. In most of these reports, there was an improvement in skin manifestations with normalization of calcemia by means of the administration of calcium and vitamin D.

In this report, this relationship was clearly demonstrated in patient 1. He presented remission of skin lesions only after the introduction of treatment for hypocalcemia. Although he did not maintain normal calcium levels due to inadequate treatment compliance, he did not present recurrent psoriasis. In contrast, patient 2 showed no improvement of skin manifestations after treatment for hypocalcemia. Although this fact may be due to the inadequate metabolic control, secondary to poor treatment compliance, it is also possible that distinct forms of psoriasis may be related to different responses to treatment of metabolic disturbances.

Other evidence that mineral metabolism abnormalities may be implicated in the pathogenesis of psoriasis has been obtained through the demonstration in some studies of reduced levels of vitamin D metabolites in psoriatic patients.^2,3^ Therefore, it is possible that hypocalcemia and/or decreased calcitriol levels are involved in the occurrence of psoriasis in pseudohypoparathyroidism, justifying the association described here. However, this association in two siblings indicates the possibility of common genetic abnormalities in the two diseases.

Although it is unlikely that the association reported here is a random occurrence, no evidence has so far been found for a relationship between the genetic abnormalities described for psoriasis and pseudo-hypoparathyroidism. Family segregation of psoriasis has been well defined. It has been suggested that this disturbance is an inherited disease with variable penetration, although it is believed that environmental factors also play a role in its clinical expression.

Several psoriasis susceptibility loci have been mapped (6p21.3, 17q, 4q, 1cen-q21, 3q21, 19p, 16q and 20p).^15^ In turn, Ga_S_protein is encoded by a gene (GNAS1) located in chromosome 20q13.1-13.2.^12^ Thus, the possibility that the association between psoriasis and hypoparathyroidism is related to other mutations not yet characterized for these diseases cannot be excluded.

In conclusion, this description points to the possible benefit of osteometabolic evaluation, at least in patients with severe forms of psoriasis. However, considering that this is the first familial demonstration of psoriasis associated with pseudohypoparathyroidism, further investigations will be needed for determining the relationship between these diseases. Prospective studies on this subject may better define the involvement of mineral metabolism abnormalities in the pathogenesis of psoriasis.
